# Lamp Shades Poisonous

**Published:** 1873-03

**Authors:** 


### LAMP SHADES POISONOUS.

Green glazed lamp shades contain arsenic and sugar of lead ; the heat reduces it in time to an impalpable powder which the slightest breath or wind detaches into the atmosphere, when it is breathed into the lungs and is at once conveyed into the circulation, giving at once a variety of disagreeable symptoms to those who habitually sit around such shades, which symptoms will promptly disappear if the shades are removed.

